# Oxidized low density lipoprotein, stem cells, and atherosclerosis

**DOI:** 10.1186/1476-511X-11-85

**Published:** 2012-07-02

**Authors:** Hui Yang, Ahmed Salah Salem Mohamed, Sheng-hua Zhou

**Affiliations:** 1 Department of Cardiology, Second Xiangya Hospital, Central South University, Changsha, Hunan, 410011, China

**Keywords:** Oxidized LDL, Stem cells, Atherosclerosis, LOX-1, Oxidative stress

## Abstract

Oxidized low density lipoprotein (ox-LDL), a risk factor of atherosclerosis, facilitates the formation and vulnerability of atherosclerotic plaque, thus contributing to several clinical complications. Stem cells participate in vascular repair after damage and atherosclerosis is a process of inflammation accompanied with vascular injury. Researchers have proposed that stem cells participate in the formation of atherosclerotic plaque. Also, because ox-LDL is capable of inducing toxic effects on stem cells, it is reasonable to postulate that ox-LDL promotes the progress of atherosclerosis via acting on stem cells. In the present article, we review the relationship between ox-LDL, stem cells, and atherosclerosis and a portion of the associated mechanisms.

## Introduction

Atherosclerosis and its main form coronary artery disease (CAD), leads to many fatalities in developed countries. Serum total and low density lipoprotein cholesterol (LDL-C) are among the firmly established risk factors of CAD [1]. Lipid lowering therapies have shown to reduce the occurrence of clinical events [2]. After oxidation, LDL becomes more toxic and plays a primary role in the development and progression of atherosclerosis [3]. The plasma levels of oxidized low density lipoprotein (ox-LDL) increased in CAD patients and increased even higher in patients with acute coronary syndrome [4,5]. Over the last few decades, Stem cells have been found as an active participator in atherosclerosis. Moreover, ox-LDL can affect stem cells in different aspects. In the present article, we dedicated to review the relationship between ox-LDL, stem cells, and atherosclerosis and part of the underlying mechanisms associated with toxic effects of ox-LDL on stem cells.

### Stem cells, an active participator in atherosclerosis

Stem cells are a group of undifferentiated, primitive cells which have the capacity of self-renewing, proliferation and differentiation into more specialized cells, such as those of the vessel, bone, heart, muscle and kidney. The offspring of stem cells are nominated as progenitor cells, which are also called, to some extent, stem cells because of their transdifferentiation. For example, EPCs could undergo SMCs drift [6].

The roles of stem cells in atherosclerosis have been summarized in a recent review [7]. In short, stem cells participated in vasculogenesis and angiogenesis, in both embryonic and post natal conditions. Also, they contribute to the repair of damaged vessels [8]. Combined with the observation that endothelial damage is the first step during atherosclerotic plaque formation, it is reasonable to postulate that stem cells are included in this procedure. Existing data also suggests that stem cells participated in the progress of atherosclerosis [9] and the extents are associated with the number of stem cells.

According to their derivation and differentiation potential, stem cells can be further divided into the following subtypes: embryonic stem (ES) cells, induced pluripotent stem (iPS) cells, mesenchymal stem cells (MSCs), and progenitor cells. Among them, ESCs are derived from the inner cell mass of a developing blastocyst and are totipotent, which possess the capacity to differentiate into cells of all 3 germ layers.

Recently, Researchers have found that forced expressions of four transcription factors c-Myc, octamer-binding transcription factor 4 (Oct4), Sox2, and Klf4 in adult germ cells were enough to generate induced pluripotent stem (iPS) cells [10]. C-Myc is a proto-oncogene which regulates cell transformation and proliferation in both transcriptional and non-transcriptional methods and is frequently deregulated in human cancers [11,12]. OCT4, also known as OCT3, belongs to the POU (Pit-Oct-Unc) transcription factor family [13]. It can activate the expression of its target genes through binding the octameric sequence motif with an AGTCAAAT consensus sequence. The expression of this gene is essential for the maintenance of pluripotentiality in ESCs and is down-regulated in all differentiated cells [14,15]. Sex determining region Y-box 2 (SOX2) is an embryonic stem cell transcription factor which expresses in human melanoma where it is associated with dermal invasion and primary tumor thickness [16]. Klf4 belongs to the Klf family which are DNA-binding transcription factors that form a subset of Cys2-His2 (C2H2) zinc-finger proteins[17]. The iPSCs are very similar to ES cells which can differentiate into all three germ layer cells.

MSCs are multipotent cells and could be derived from different kind of adult tissue, such as bone marrow, adipose tissue, artery wall and so on. Under certain conditions, they have the ability to give rise to all three germ layer cells, especially cardiomyocytes, endothelial cells, smooth muscle cells, etc. [18,19]. In particular, because of their hypoimmunity, facilitated derivation and culture in vitro, and potency to autologous transplantation, they are thought to be an ideal donor for cells in tissue regeneration [20]. Bone marrow derived MSCs can enter the systemic circulation and migrate into vascular and other connective tissues, especially at sites of injury and in tissue transplant grafts. Transplantation of MSCs is beneficial to limb ischemia patients [21]. Moreover, MSCs exert a paracrine capacity through the release of cytokines [22], and regulation of the WNT pathway (WNT pathway has been implicated in the self-renewal and maintenance of pluripotent stem cells and progenitor cells [23,24]) was thought to be one of its main mechanisms in cardiac repair after myocardial infarction. Notably, the role of MSCs after transplantation seems largely influenced by the microenvironment and the local factors. For example, hepatocyte growth factor (HGF,also known as hepapoietin A, can be produced by various cells and elicits multiple biological responses such as motility, proliferation, morphogenesis and survival in a cell type-dependent fashion [25]) from atherosclerotic plaque derived smooth muscle cells (SMCs) plays an important role for the homing MSCs to differentiate into smooth muscle cells, which may contribute to the deterioration of atherosclerotic plaque [26]. Paradoxically, MSCs seeding can inhibit the proliferation and migrations of SMCs. MSCs co-cultured with mature endothelial cells (ECs) have the ability to undergo milieu-dependent differentiation toward ECs [27]. Migrated MSCs from bone marrow may play a key role in the development of atherogenesis in response to ox-LDL, which could induce MSCs derived smooth muscle like cells to differentiate into foam cells [28,29].

Progenitor cells, also known as unipotent stem cells, which are derived from more developed fetal or adult tissues, are able to generate more restricted lineages than ESCs and MSCs. For example, endothelial progenitor cells (EPCs), which could differentiate into endothelial cells, would participate in vascular repair and contribute to the integrity and function of endothelial membrane [30]. Compared with EPCs, the roles of smooth muscle progenitor cells (SMPCs) in atherosclerosis seem to be more complicated. While increased number of SMPCs in the plaque contributed to the severity of luminal stenosis, decreased amount of SMPCs account for a thinner neointima and plaque instability [31]. Interestingly, Fadini and coworkers [6] reported that the precursors of EPCs and SMPCs seem to be the same, and EPCs could undergo SMCs drift; thus, EPC dysfunction, may be compensated by an increase in SMPCs.

### Oxidized low density lipoprotein and atherosclerosis

Lipoprotein plays key role in atherogenesis. It transport lipids, such as cholesterol and triglycerides, in the circulation. The intestine uptakes fat from diet and packages it into chylomicrons (large triglyceride-rich lipoproteins). The latter is then transported to peripheral tissues via the blood. Inside muscle and adipose tissues, the enzyme lipoprotein lipase breaks down chylomicrons, and fatty acids enter these tissues. The chylomicron remnants are subsequently taken up by the liver. The liver loads lipids onto apoB and secretes very-low-density lipoproteins (VLDLs), which undergo lipolysis by lipoprotein lipase to form low-density lipoproteins (LDLs) [32]. ApoB is one of the main vectors for lipids transportation and plays a central role in the composition of LDLs. LDL is essential for normal cellular function, but in high concentrations, especially after oxidized or carbamylated [30,33], it would facilitate atherosclerotic plaque formation. Over the past several decades, it has been proved that the oxidized form of LDL is more important in the genesis and progression of atherosclerosis than native unmodified LDL. The serum concentration of ox-LDL elevated in stable coronary artery disease (CAD) patients and became even higher in acute coronary syndrome (ACS) ones [4,5,34,35]. Low density lipoprotein oxidation is a progressive process which leads to the formation of mildly to extensively oxidized LDL. The ox-LDL contains various toxic oxidized lipids in different proportions [36]. The toxic components of ox-LDL may include aldehydes, oxysterols, lipid peroxides and so on. One of the best characterized changes during oxidation is the affinity of ox-LDL to target cells. In the process of oxidation, the apoB in LDL changed, which led to the affinity of ox-LDL to apoB receptors to decrease as the affinity to the scavenger receptors, such as CD36 and lectin like oxidized low density lipoprotein receptor-1 (LOX-1), increased [36]. (CD36 belongs to the Class B Scavenger Receptors, which are characterized by the presence of membrane-spanning N and C termini and a large extracellular loop [37]. LOX-1 is a type II membrane protein with a short cytoplasmic tail and extracellular domain. Structurally it belongs to the C-type lectin family, which could bind to carbohydrates in a Ca^2+^-dependent manner, and is comprised of four domains: a short N-terminal cytoplasmic domain, a single transmembrane domain; a short ‘neck’ or stalk region and a C-type lectin-like fold [38]). In 1987, Kita and coworkers investigated the proatherogenic role of ox-LDL and the therapeutic effects of antioxidant agent probucol. They found that probucol could prevent the progression of atherosclerosis in homozygous Watanabe heritable hyperlipidemic rabbits (an animal model for familial hypercholesterolemia) in vivo by limiting oxidative LDL modification and foam cell transformation of macrophages [39]. Besides oxidative stress, ox-LDL could induce endothelial cell apoptosis through different methods, which may include signal transduction, gene expression, and calcium signal system. Now, it is well known that ox-LDL–induced toxic effects are present in all stages of atherosclerosis from beginning to the acute thrombotic events [40]. Ox-LDL also contributs to the rupture of fibrous cap via promoting the secretion of matrix metalloproteinases (MMPs), which are a family of zinc-dependent endopeptidases and mediate the degradation of protein components of the extracellular matrix and of basement membranes in target cells [41], and promoting formation of platelet clots in narrowed arteries which could cause myocardial infarction. Not only circulating ox-LDL concentration, but also the increased levels of ox-LDL in human coronary atherosclerotic lesions are related to plaque instability. Also, the more severe lesions contain a significantly higher percentage of ox-LDL-positive macrophages [42].

### Oxidized low density lipoprotein and stem cells

**Figure 1:**
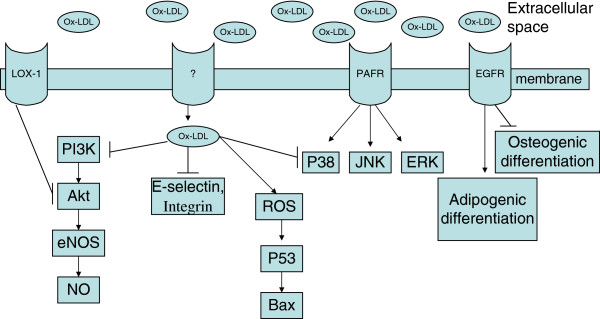
**The molecular events included in the direct toxic effects of ox-LDL on stem cells.** ?: whether receptors mediate phagocytosis of ox-LDL in MSCs is not clear; →: positive effects; ⊥: negative effects.

LDLs in the blood could enter the intima, where they are retained through binding to the extracellular matrix. LDLs are then modified by oxygen radicals, myeloperoxidase, secretory phospholipase A2 and sphingomyelinase [32]. The rate of oxidants liberated by cultured cells and their ability to oxidize LDL is largely variable and is dependent on species, cell type, proliferation rate, and culture medium [36]. Monocyte-derived macrophages and neutrophils are able to initiate and propagate lipid peroxidation [43]. To the best of our knowledge, whether stem cells could modificate LDLs and/or the involved mechanisms are not clear. Even if stem cells have no effect on LDL oxidation, the LDL oxidized by other cells, such as monocytes, may contribute to the participation of stem cells in the process of atherosclerosis. However, it is important to know the direct effects of stem cells on LDL and its role in atherogenesis, which would let us to know whether stem cells can initiate atherosclerosis without the help of other cell types. Systemic lipids from diet can be delivered to the bone marrow by chylomicron and chylomicron remnants [44,45]. Hyperlipidemia may negatively influence the stem cells in both bone marrow and systemic circulation pool. There are large number of evidences that ox-LDL could act on stem cells, especially EPCs, SMPCs, and MSCs to influence the cellular physical activities in almost every aspects, such as proliferation, differentiation, apoptosis, mobilization, migration, senescence, and so on. The associated mechanisms might include membrane receptors mediated phagocytosis and signal transduction, oxidative stress, mitogen activated protein kinase (MAPK) pathway and others, which may be further divided into direct (Figure 1) and indirect effects.

### Direct effects of ox-LDL on stem cells

#### Membrane receptors

As mentioned above, ox-LDL was able to bind to cell membrane receptors. The toxic effects of ox-LDL are mainly mediated by membrane receptors. For example, there are relationships between the extent of cytotoxicity and the amount of ox-LDL taken up by the cells through membrane receptors [46]. On the other hand, membrane receptors could intefere with cellular function through signal transduction. In fact, the former seems to be more important in introducing cellular toxicity than the latter [36]. The identified membrane receptors may include LOX-1, platelet activating factor receptor, and epithelial growth factor.

LOX-1 is associated with ox-LDL induced EPC senescence [47] and apoptosis, and declined capacities of survival, adhesion, migration and tube formation [48]. Furthermore, it contributes to the formation of foam cells derived from MSCs [29]. LOX-1 expression could be induced in pathological conditions, such as atherosclerosis [3,49]. Yu et al. [29] examined the differentiation of foam cells from MSCs after ox-LDL treatment. While different concentrations of ox-LDLs were added into the culture media, the expressions of LOX-1 were enhanced gradually as the increasing of concentrations. Furthermore, LOX-1 mediated phagocytized lipid droplets accumulated in the cytoplasm of foam cells and the number of droplets increased as LOX-1 upregulated, and, LOX-1 contributed to the ox-LDL-induced transdifferentiation of foam cells. In long term incubation of ox-LDL (10 μg/ml) with endothelial progenital cells, ox-LDL significantly accelerated the rate of senescence compared with controls. On the other hand, ox-LDL significantly diminished telomerase activity to approximately 50% compared with controls, such induced cell senescence. The proliferative capacity and network formation also could be impaired by ox-LDL and all these effects were mediated by LOX-1, as shown by the phenomenon that ox-LDL-induced EPC senescence was significantly inhibited by pretreatment with either LOX-1 antibody or atorvastatin [47]. In addition, some subtypes of ox-LDL could give rise to EPCs senescence via LOX-1/Akt pathway [50].

It has been proved that PAF-R may introduce ox-LDL induced MSC migration [28]. Ox-LDL contains platelet activating factor (PAF) and PAF-like phospholipids [51]. Activation of PAF receptor (PAF-R) is associated with oxLDL-induced chemokine release and adhesions of monocytes [52,53]. Shin et al. [28] showed that at a relatively low concentration (from 0.1-2 μg/ml), ox-LDL dose-dependently stimulated chemotactic migration of human bone marrow MSCs in vitro via PAF-R. This effect could be completely aborogated by PAF-R-specific antagonist BN52021 at a concentration of 10 μM or blocked by siRNA-mediated depletion of endogenous PAF-R expression.

Epithelial growth factor receptor(EGFR), a receptor tyrosine kinase, has been proved to exist in MSCs [54]. Minimally oxidized low density lipoprotein (MM-LDL) or high fat diet was able to promote osteoporotic loss of bone by directing progenitor marrow stromal cells to undergo adipogenic instead of osteogenic differentiation. The inhibitory effect of MM-LDL on alkaline phosphatase (a marker of osteoblastic differentiation) activity in murine marrow stromal cells could be mimicked by pretreating cells with 50 ng/ml or 100 ng/ml of EGF [55]. This phenomenon indirectly suggested that ox-LDL and EGF may have same membrane receptors in introducing their biologic effects on stromal cells.

Taken together, Binding to the membrane receptors seem to be the first step in ox-LDL’s toxic effects and mediate the phagocytosis of ox-LDL. Then, after arriving in the cytoplasm, ox-LDL could generate oxidative stress, stimulate MAPK signal transduction, and change the target genes’ expressions to introduce a large number of toxic effects [36].

#### Oxidative stress

Ox-LDL could impair MSC proliferation through oxidative stress [56,57]. Meanwhile, the modified LDL could induce EPCs apoptosis (programmed cell death) and senescence (irreversible cell-cycle arrest), and impair cell proliferation, migration and neovasculorization via oxidative stress, which were identified by the facts that cellular reactive oxygen species (ROS) concentration increased, mitochondrial transmembrane potential decreased, and cytochrome-c oxidase expression increased after cells were treated with ox-LDL. [30,58]. Other proofs were that the antioxidant agents, such as N^G^-nitro-L-arginine methyl ester (L-NAME) (L-NAME is an L –arginine analogue that inhibits all nitric oxide synthase isoforms. Oxidative stress uncouples endothelial nitric oxide synthase by oxidation of the essential co-factor tetrahydrobioptein, so that endothelial nitric oxide synthase activity generates O_2_^-^, not NO [59]), epicatechin, Danshen, Danshensu, superoxide dismutase (SOD) (SOD is an enzyme that dismutates O_2_^-^ to H_2_/O_2_), statin, and even red wine consumption all could protect stem cells from the toxic effects of ox-LDL [9,58,60-62]. High concentration of ROS induced by ox-LDL was able to activate P53 (a tumor suppressor which can induce different cellular outcomes such as cell cycle arrest, senescence and apoptosis), and promote the translocation of Bax, which belongs to the anti-apoptotic Bcl-2 family proteins, to mitochondria, such induce EPC apoptosis. Intriguingly, some parts of oxidized LDL were capable of improving superoxide dismutase (SOD) concentration, which could protect EPCs from high concentration of ox-LDL induced apoptosis [63]. Oxidative stress also mediated the ox-LDL inhibited MSCs proliferation, as demonstrated by the fact that the expression of stem cell marker OCT-4 was downregulated, and oxidative stress contributed to blocked endothelial differentiation of these cells [57]. The anti-oxidant reagent, N-acetylcysteine, could antagonize these toxic effects, and these roles are independent to Akt expression. On the other hand, there are crosstalk between oxidative stress and PI3K/Akt pathway. For example, it has been shown that ox-LDL was associated with increased O^2-^ and H_2_O_2_ concentration in target cells and was able to induce a dose- and time- dependent reduction to the p-Akt/Akt ratio and to increase in apoptotic rate [58]. These effects were significantly reduced by the antioxidants SOD and L-NAME. The intracellular oxidative stress suppressed the PI3K/Akt/endothelial nitric oxide synthase(eNOS)/nitric oxide(NO) pathway in EPCs [58,64].

#### PI3K/Akt pathway

Inhibited Phosphoinositide 3-kinase (PI3K) pathway contribute to impaired EPC differentiation, mobilization, migration and proliferation [65-67]. It also contribute to ox-LDL induced MSC apoptosis [56,57]. PI3Ks are a family of enzymes involved in cell growth, proliferation, differentiation, and so on. Akt serves as a multifunctional regulator of cell biology [68]. Phosphorylated Akt becomes available to phosphorylate its downstream substrates, such as endothelial nitric oxide synthase (eNOS), leading to eNOS activation and nitric oxide (NO) release [48,69]. The pleckstrin homology domain of AKT can bind immediately to PtdIns(3,4,5)P3 and PtdIns(3,4)P2, which are generated by activated PI3k [70]. Ox-LDL could inhibit vascular endothelial growth factor induced EPCs differentiation via depressing PI3K/Akt pathway. Immunoblotting analysis revealed that ox-LDL dose-dependently led to dephosphorylation and, deactivation of Akt in the presence of VEGF. Notably, these inhibitory effects induced by ox-LDL were abolished by pretreating cells with 1 μmol/L atorvastatin [65]. Besides differentiation, ox-LDL depressed PI3K/Akt/eNOS/NO pathway contributed to decreased EPC proliferation, migration, and cell number decreasing [66,67]. The stimulation of NO production or its signal cascades in EPCs may increase their number and improve their function, thus attenuate endothelium damage. These effects were independent of the vasodilatory effects of NO [71] but the antioxidant effects. Interestingly, it is Akt depressing which mediated the inhibitory effects of ox-LDL on MSCs at a concentration higher than 5 μmol/L, but not oxidative stress as mentioned above [57]. Akt overexpression in MSCs transfected with a constitutively active Akt completely reversed the toxic effects of ox-LDL on MSCs, such as apoptosis, decreased cell proliferation, suppressed Oct-4 expression and endothelial differentiation as well as in vitro vascular structure formation [56].

#### MAPK pathway

MAPKs mediate MSC migration[28] and EPC proliferation [72]. MAPKs are serine/threonine-specific protein kinases which belongs to the CMGC (CDK/MAPK/GSK3/CLK) kinase group [73]. MAPKs are involved in modulating cellular responses to a diverse series of stimuli, such as mitogens, osmotic stress, heat shock and proinflammatory cytokines. Under physical or pathologic conditions, They regulate cell proliferation, differentiation, mitosis, gene expression, cell survival, and apoptosis [74]. Three MAPKs have been found: P38, Jun amino-terminal kinase (JNK) and extracellular-signal-regulated kinases 1/2 (ERK1/2). Oxidized LDL induced MSC migration seems to be associated with all these three MAPKs, and cell migration could be abrogated by pretreating cells with either MAPK inhibitors [28]. The p38 MAPK also plays a critical role in regulating the number and functions of EPCs in vitro. SB203580, an inhibitor of the p38 MAPK, can improve the number and functions of EPCs under basal conditions and prevent the negative effects of oxLDL on the number and functions of EPCs [72].

#### Other mechanisms

It has been reported that incubation of EPC with sub-apoptotic ox-LDL concentrations significantly decreased E-selectin (a cell adhesion molecule expressed only on endothelial cells activated by cytokines) and integrin α_V_β_5_ (integrin α_V_β_5_ belongs to the integrin family which are receptors that mediate the attachment between a cell and the tissues that surround it, such as other cells or the extracellular matrix.) expression, which led to inhibited tube formation and integration. Blocking of E-selectin and integrin α_V_β_5_ by neutralizing antibodies effectively inhibited adhesion of EPC to differentiated endothelial cells and facilitated the tube formation in vitro [75].

### Indirect effects of ox-LDL on stem cells

Theoritically speak, aside the direct effects of ox-LDL on stem cells, ox-LDL may act on other cell types to indirectly influence the function and number of stem cells. These cell types may include monocyte/macrophage [76-78], leukocyte subsets [79], endothelial cells [79], platelet [80], and so on. For example, Rohde et al. found that endothelial colony-forming unit formation (CFU-EC) by EPCs was strictly dependent on monocyte presence, though neither intrinsic nor cultured monocytes formed vascular networks. The authors speculate that monocytes or monocyte-derived regulatory factors may be peculiar in the generation of CFU-EC [81], but the exact mechanism is not clear.

The contributions of leukocyte subsets to atherosclerosis in mouse models were fully reviewed by Weber et al. in a recent article [79]. Briefly speak, the authors focused on the contributions of granulocyte subsets and mast cells to early atherogenesis and subsequent plaque instability, and the role of monocyte, macrophage and dendritic-cell subsets is heterogeneous and double-edged. There are interaction between these cells and vascular progenitor cells. Macrophage can excrete matrix metalloproteinases-9 (MMP-9), which mediates extracellular matrix degradation and are expressed in atherosclerotic plaque. MMP-9, under certain conditions, combined with CXCL12/CXCR4 (CXC-chemokine receptor 4 and its ligand CXC-chemokine ligand 12, also known as stromal cell-derived factor 1α) mediate the migration of bone marrow stem cells from the bone marrow stroma [82]. Also, injured SMCs and endothelial cells express CXCL12, which would bind to its receptor CXCR4 to trigger the arrest of progenitor cells at sites of arterial injury [83], and benefit to neovascularization. This was confirmed by the fact that cell migration could be totally inhibited by AMD3100, a CXCR4-specific peptide antagonist. The migration can also be nearly completely blocked by PI3K inhibitors (LY294002 and wortmannin) and eNOS inhibitor (N-nitro-arginine methyl ester) [84], which indicate that there is crosstalk between PI3K pathway and CXCL12/CXCR4. Platelets mediate the transmigration of monocytes into the vessel wall. While uptake the ox-LDL, ox-LDL laden platelets would induce foam cell development from CD34+ progenitor cells, and the number of CD34+ progenitor cells (colony-forming units) able to transform into endothelial cells was significantly reduced in the presence of ox-LDL-platelets [85].

### Synergistic toxic effects with hyperglycemia

The facts that patients with diabetes mellitus had higher rate of cardiovascular diseases gave rise to the researches about the toxic effects of hyperglycemia on cells in atherosclerosis. Now it’s clear that hyperglycemia (HG) alone or combined with hypercholesterolemia has synergistic toxic effects on the survival and function of stem cells [86]. Hamed et al. revealed that the NO system might contribute to the dysfunction and metabolic alterations of EPCs in diabetes mellitus type-2(DM-2). Kra¨nkel et al. [67] reported that HG conditions could cause a significant decrease in healthy EPCs. Furthermore, HG conditions could also impair cell’s migrational and integrative capacities. These effects may be mediated by the enhanced Akt counterplayer protein phosphatase 2A activity in circulating progenitor cells, which resulted in a declined eNOS phosphorylation at Ser^1177^ and, NO liberation. Otherwise, the toxic effects of hyperglycemia alone seem to be insufficient to cause severe vascular complications in DM-2 patients. Fadini et al. proposed that a combination of hyperglycaemia and other cardiovascular risk factors, such as hyperlipidaemia, could most likely explain the serious reduction in circulating EPC counts in DM-2 patients [87].

Taken together, the toxic effects of ox-LDL on stem cells are multifaceted and complicated which may include both direct and indirect ones. As binding to the membrane receptors is the first step of ox-LDL to directly act on stem cells, and it is postulated that involvement of the scavenger receptors in the toxicity may be more related to their roles in ox-LDL uptake than to a direct toxic cell signaling [36]. It is logic to speculate the membrane receptors may play key roles in introducing the effects of ox-LDL on stem cells. However, compared with the intracellular mechanisms, the existed researches about membrane receptors mediated toxic effects of ox-LDL on stem cells are very limited. Further studies on this issue need to be extensively explored. Compared with the direct effects of ox-LDL on stem cells, the indirect effects are poorly investigated. Under the local microenvironment of a plaque, the crosstalk between different cell types and stem cells may be of important value and deserve to be fully studied, which may benefit to the promotion of our knowledge on the atherogenesis. Moreover, the Synergistic toxic effects of coronary risk factors such as hypertension, cigarette smoking, hyperlipidemia, hyperglycemia need to be further studied.

## Statin act as an excellent therapeutic strategy to ox-LDL induced toxic effects

HMG-CoA reductase inhibitors (statins) have been developed as lipid-lowering drugs and have revolutionized the treatment of hypercholesterolemia. These drugs are well established to reduce morbidity and mortality for coronary artery disease patients. Besides lipid regulation, statins are able to reduce vascular inflammation, alleviate platelet aggregation and thrombus deposition, and enhance endothelium-derived nitric oxide production [88]. As mentioned above, researchers have declared that statins got therapeutic effects to nearly all the ox-LDL induced toxicities on stem cells. Imanishi et al. reported that atorvastatin pretreatment was capable to antagonize oxidized LDL-induced EPC senescence and impaired net work formation, and these effects were similar to the LOX-1 antibody [47]. Statins could stimulate the PI3K/Akt pathway, thereby inducing EPC mobilization [89,90]. Hamed et al.[91] have shown that statins are associated with NO bioavailability in diabetic EPCs. It has also been proved that statins could increase the EPC count in patients with CAD [92]. Moreover, statins and SDF1α could synergistically enhance EPC proliferation, and MMP-2, 9 expressions by activating the Akt/NOS pathway. Also, the activation of this pathway by statin contributed to the restoration of function of EPCs damaged by ox-LDL [93,94]. Recently, Hamed and colleagues demonstrated that simvastatin can stimulate CXCR4 expression and PI3k/Akt/eNOS signaling pathway in EPCs exposed to simultaneous HG and OxLDL, and the authors proposed that statin therapy may be beneficial for type 2 DM patients with CAD through promoting neovascularization and the formation of a coronary collateral circulation [86]. In hypertensive hypercholesterolemic pigs, statin rescued renal repair through inhibiting local EPC apoptosis [95]. Interesting, statin may have the ability similar to VEGF in promoting circulating monocytes and CD34^+^ hematopoietic stem cells differentiation into EPCs via the PI3K/Akt pathway [89].

## Conclusion

To the best of our knowledge, this is the first review about the effects of ox-LDL on stem cells. Ox-LDL, a risk factor of coronary artery disease, contributed to the formation and instability of atherosclerotic plaque partly through the decreasing number and dysfunction of stem cells which exposed to ox-LDL. Compared with EPCs, the toxic effects of ox-LDL on other stem cells such as MSCs and the associated mechanisms have not been fully investigated. Stem cells, for example, MSCs, are active in several restoration processes, such as restoration of injured endothelium, replacement of the damaged cardiomyocytes, and beneficial paracrine effects after myocardial infarction. Otherwise, the roles of ox-LDL on MSC differentiation besides endothelial differentiation, mobilization, senescence are unclear. Either, the toxic effects of hyperglycemia alone or combined with hypercholesterolemia on MSCs are unclear. Understanding the toxic effects of ox-LDL on MSCs and the underlying mechanisms may contribute to the discovery of specialized antagonist, and this is meaningful, especially to the patients whose serum lipid was poorly controlled or the patients who had both CAD and diabetes mellitus. Statin may act as a possible therapeutic strategy among the possible choices.

## Abbreviations

ACS: Acute coronary syndrome; CAD: Coronary artery disease; CFU: Colony forming unit; CXCL: CXC-chemokine ligand; CXCR: CXC-chemokine receptor; EC: Endothelial cell; EGF: Epithelial growth factor; EGF-R: EGF receptor; eNOS: Endothelial nitric oxide synthase; EPC: Endothelial progenitor cells; ERK: Extracellular signal regulated kinase; ESC: Embryonic stem cells; HGF: Hematocyte growth factor; HSC: Haematopoietic stem cells; iPS: Induced pluripotent stem cells; JNK: JUN Amino-terminal kinase; LDL-C: Low density lipoprotein cholesterol; Lox-1: Lectin like oxidized low density lipoprotein receptor 1; LDL: Low density lipoprotein; L-NAME: NG-nitro-L-arginine methyl ester; MAPK: Mitogen activated protein kinase; MMP: Metalloproteinase; MM-LDL: Minimally oxidized low density lipoprotein; MSC: Mesenchymal stem cells; NO: Nitric oxide; OCT4: Octamer-binding transcription factor 4; ox-LDL: Oxidized low density lipoprotein; PAF: Platelet activating factor; PAF-R: PAF receptor; PI3K: Phosphoinositide 3 kinase; ROS: Reactive oxygen species; SDF: Stormal cell derived factor; SMC: Smooth muscle cell; SMPC: Smooth muscle progenitor cells; SOD: Superoxide dismutase; VLDL: Very low density lipoprotein.

## Competing interests

The authors declare that they have no competing interests.

## Authors’ contributions

HY, ASM and SHZ conceived the study, its design and drafted the manuscript. All authors read and approved the final manuscript.
